# Supplementary material for: An integrative analysis of genomic and exposomic data for complex traits and phenotypic prediction
Source: Sci Rep. 2021 Nov 2;11:21495. doi: 10.1038/s41598-021-00427-y (PMC8564528; doi:10.1038/s41598-021-00427-y)
Supplement: Supplementary file 1 — Supplementary Information. [file 41598_2021_427_MOESM1_ESM.docx]

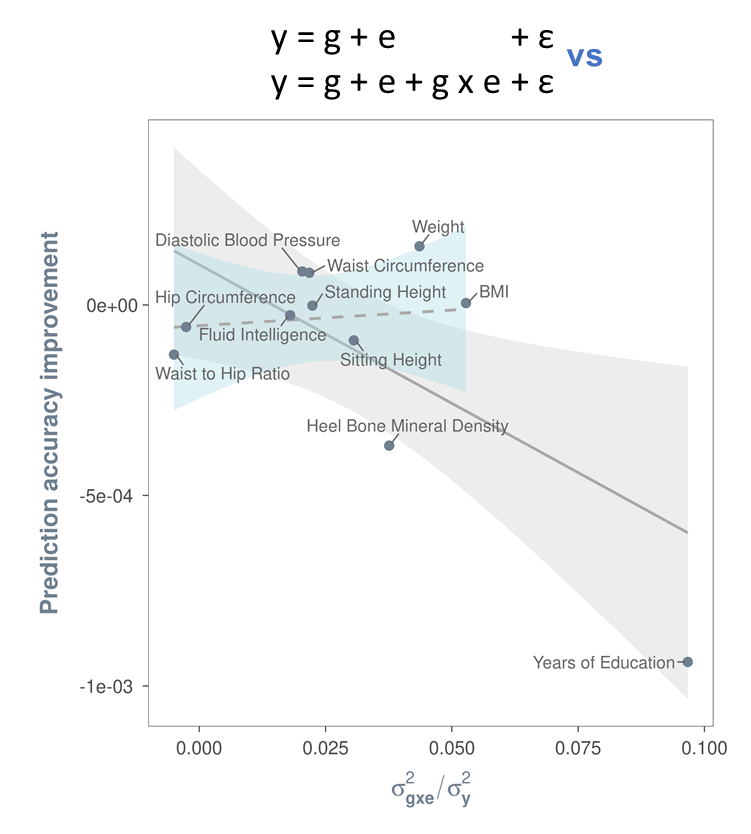


# Supplementary Figure 1. Interactions between SNP genotypes and exposomic variables (gxe interactions) contribute to phenotypic variance but not phenotypic prediction accuracy. $\sigma_{\mathrm{gxe}}^{2}$ denotes the phenotypic variance explained by gxe interactions. Prediction accuracy was computed using the Pearson’s correlation coefficient between the observed and the predicted for models with and without a random term for phenotypic effects of gxe interaction, denoted as y = g+e+ε and y = g+e+gxe+ε, respectively. g = phenotypic effects of the genome; e = phenotypic effects of the exposome; ε = residuals. Prediction accuracy improvement (i.e., y-axis) was derived by subtracting the prediction accuracy of the model y = g+e+ε from that of the model y = g+e+gxe+ε. Least squares lines with 95% confidence band are based on a linear model that regressed prediction accuracy improvements on estimates of variance explained by gxe interactions. The solid line is based on all traits, and the dashed line is based on traits without years of education. The slope of the solid line is statistically different from zero (t(7)=0.02), but the slope of the dashed line is not (t(6)=0.78).


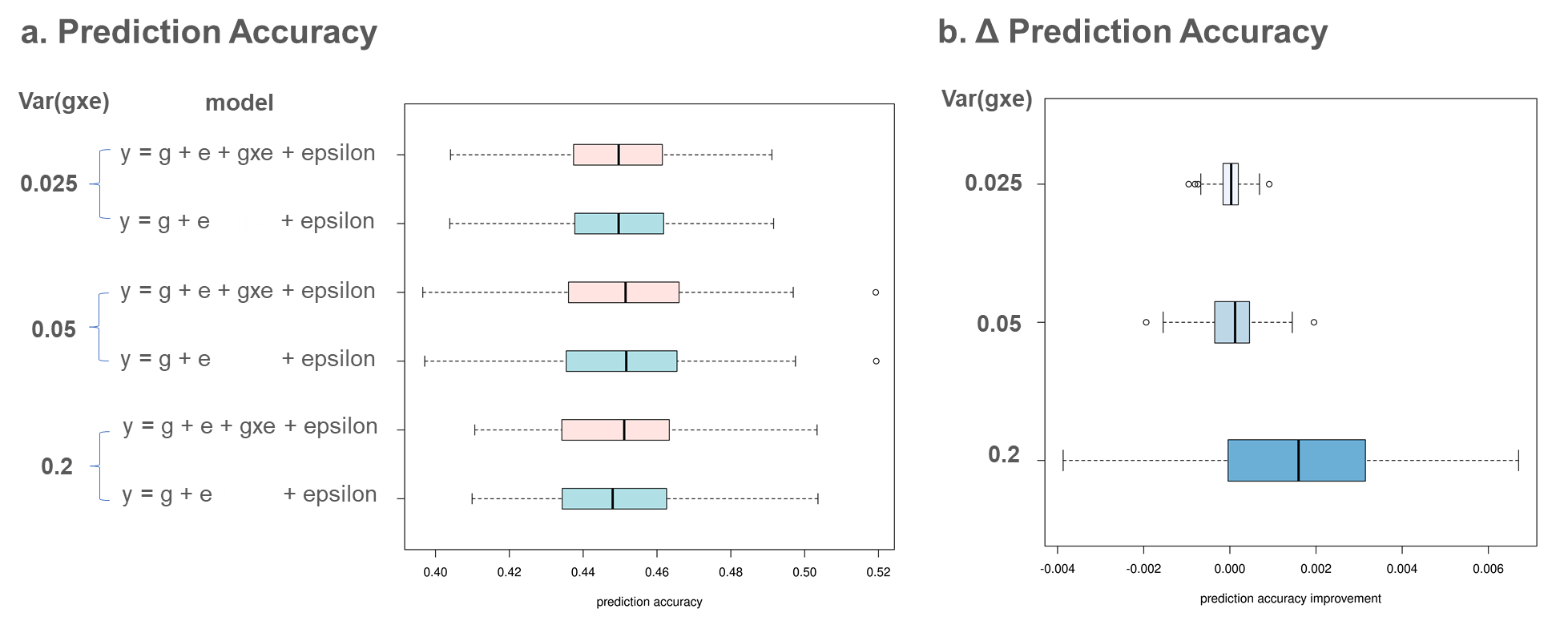


# Supplementary Figure 2. Simulation results showing that despite the presence of genuine gxe interaction effects, little phenotypic prediction accuracy can be gained from accounting for these interactions.

#
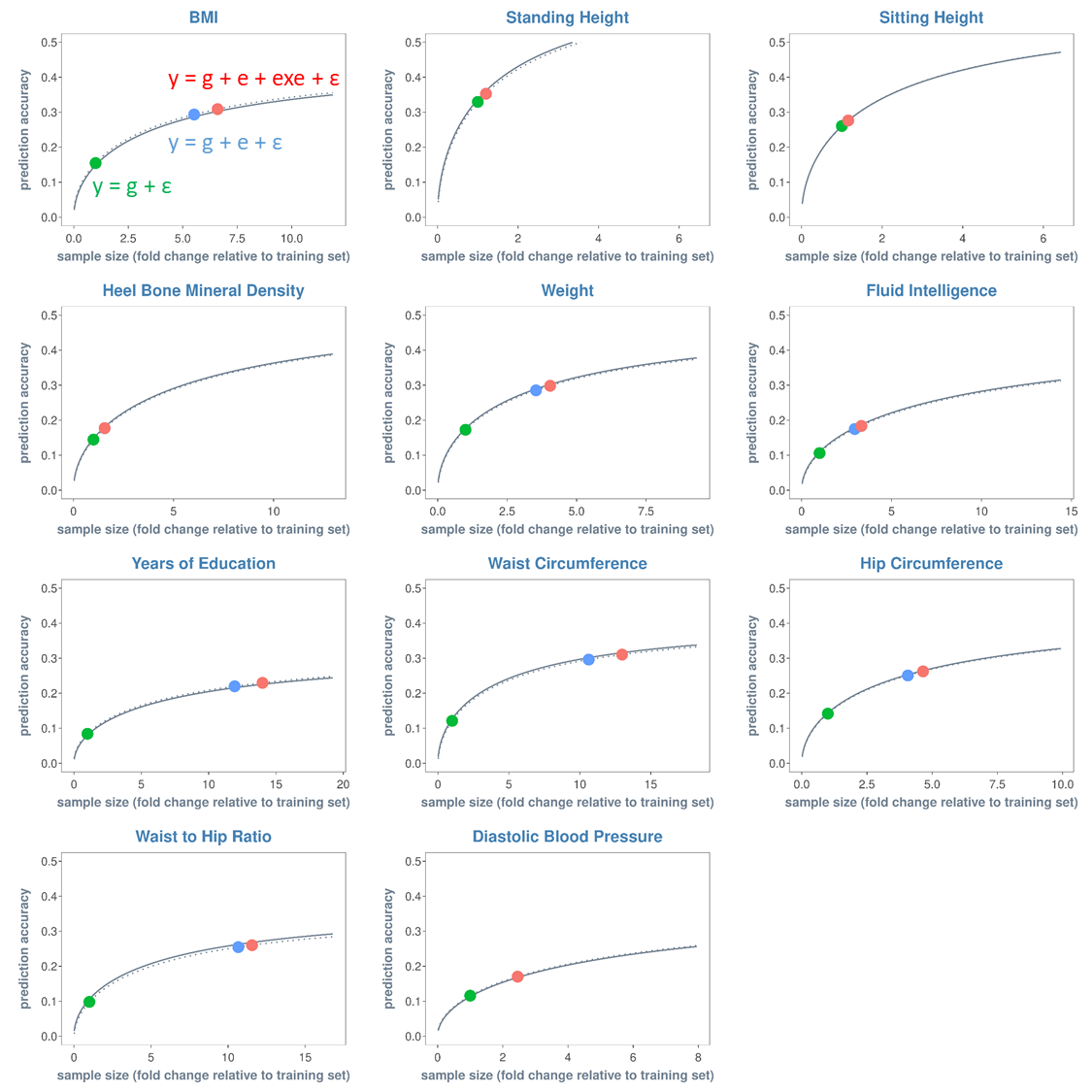
Supplementary Figure 3. Additional sample size required for the model y = g + ε to achieve the same level of prediction accuracy as y = g + e + ε (blue) and y = g + e + exe + ε (red). Sample size is expressed relative to that of the training (or discovery) dataset for each trait. Each solid curve represents the projected prediction accuracy as a function of sample size for the model y = g + ε using established theories (see Methods). Each green dot represents the observed prediction accuracy by y = g + ε at the given sample size. Dotted curves are prediction accuracies adjusted for the difference between the theoretical prediction accuracy and observed prediction accuracy, noting that the difference is minimal for all traits such that most dotted curves are obscured by the solid curve. The y-coordinates of the blue and red dots correspond to the observed prediction accuracies for models y = g + e + ε and y = g + e + exe + ε, respectively. The x-coordinates of the blue and red dots correspond to the projected sample sizes required for y = g + ε to achieve the same level of prediction accuracy as y = g + e + ε and y = g + e + exe + ε.


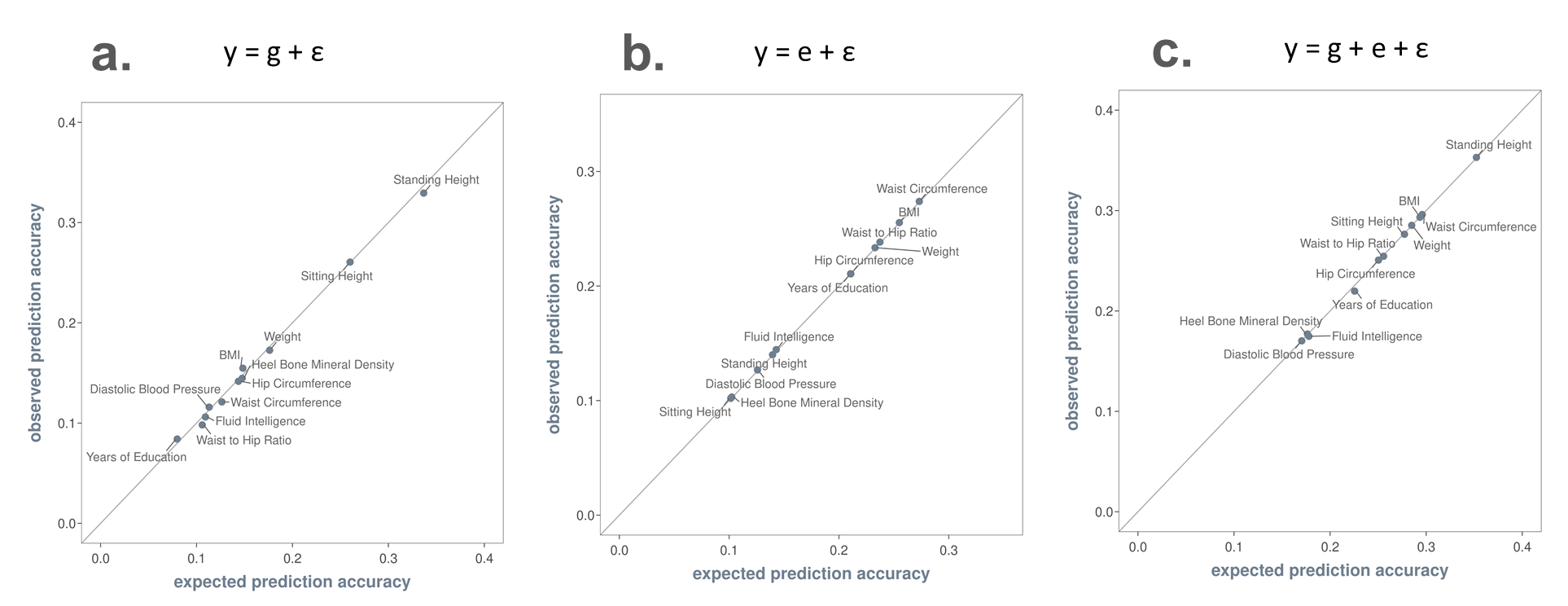


# Supplementary Figure 4. Prediction accuracies derived from theories agree with prediction accuracies based on the real data. Expected prediction accuracies are based on established theories (see Methods). Observed prediction accuracies are based on results from 5-fold cross validations. Panel a. prediction accuracy for the model y = g + ε. Panel b. prediction accuracy for the model y = e + ε. Panel c. prediction accuracy for the model y = g + e + ε.

# Supplementary Table 1. Variance and covariance estimates from model y = g + e + ε, where $\sigma_{\mathrm{ge}}$ is a free parameter for estimation.

| **Trait** | $\sigma_{g}^{2}$ | | $\sigma_{e}^{2}$ | | $\sigma_{\mathrm{ge}}$ | |
| --- | --- | --- | --- | --- | --- | --- |
|  | est. | s.e. | est. | s.e. | est. | s.e. |
| bmi | 1.6E-01 | 8.9E-03 | 5.2E-02 | 1.4E-02 | 2.9E-03 | 3.5E-02 |
| height | 5.0E-01 | 1.2E-02 | 1.5E-02 | 4.2E-03 | 3.5E-02 | 2.0E-02 |
| sitheight | 3.3E-01 | 1.0E-02 | 7.9E-03 | 2.3E-03 | 1.8E-02 | 1.4E-02 |
| heelbmd | 2.5E-01 | 1.8E-02 | 9.7E-03 | 3.0E-03 | -1.2E-02 | 2.0E-02 |
| weight | 2.0E-01 | 9.6E-03 | 4.4E-02 | 1.2E-02 | 2.1E-02 | 3.2E-02 |
| fluidiq | 2.0E-01 | 2.1E-02 | 2.1E-02 | 5.9E-03 | 2.9E-02 | 3.0E-02 |
| edu | 8.9E-02 | 9.8E-03 | 4.6E-02 | 1.3E-02 | -6.9E-02 | 3.2E-02 |
| waist | 1.5E-01 | 9.6E-03 | 6.6E-02 | 1.8E-02 | -1.7E-02 | 4.0E-02 |
| hip | 1.6E-01 | 8.9E-03 | 3.6E-02 | 9.8E-03 | 2.3E-02 | 2.8E-02 |
| wsthipr | 1.3E-01 | 1.0E-02 | 5.3E-02 | 1.4E-02 | -1.9E-02 | 3.4E-02 |
| diapres | 1.6E-01 | 1.1E-02 | 1.6E-02 | 4.4E-03 | -6.0E-04 | 1.9E-02 |

# Supplementary Table 2. Breakdown of phenotypic variance by the model y = g + e + exe + gxe + ε.

| **Trait** | $\sigma_{g}^{2}$ | |  | $\sigma_{e}^{2}$ | |  | $\sigma_{\mathrm{exe}}^{2}$ | |  | $\sigma_{\mathrm{gxe}}^{2}$ | |
| --- | --- | --- | --- | --- | --- | --- | --- | --- | --- | --- | --- |
|  | *est* | *se* |  | *est* | *se* |  | *est* | *se* |  | *est* | *se* |
| bmi | 1.93E-01 | 1.06E-02 |  | 7.25E-02 | 1.82E-02 |  | 1.88E-02 | 2.10E-03 |  | 4.52E-02 | 1.05E-02 |
| height | 5.18E-01 | 1.08E-02 |  | 1.73E-02 | 4.70E-03 |  | 4.00E-04 | 7.00E-04 |  | 2.21E-02 | 9.60E-03 |
| sitheight | 3.92E-01 | 1.10E-02 |  | 9.60E-03 | 2.70E-03 |  | 3.00E-04 | 8.00E-04 |  | 3.03E-02 | 1.03E-02 |
| heelbmd | 3.10E-01 | 2.17E-02 |  | 1.21E-02 | 3.70E-03 |  | 2.50E-03 | 1.90E-03 |  | 3.53E-02 | 1.72E-02 |
| weight | 2.37E-01 | 1.10E-02 |  | 5.93E-02 | 1.52E-02 |  | 1.60E-02 | 1.90E-03 |  | 3.68E-02 | 1.04E-02 |
| fluidiq | 2.05E-01 | 2.04E-02 |  | 2.21E-02 | 6.30E-03 |  | 1.14E-02 | 2.40E-03 |  | 1.23E-02 | 1.59E-02 |
| edu | 7.79E-02 | 9.30E-03 |  | 4.75E-02 | 1.24E-02 |  | 1.15E-02 | 1.60E-03 |  | 9.20E-02 | 1.16E-02 |
| waist | 1.59E-01 | 1.03E-02 |  | 8.47E-02 | 2.10E-02 |  | 1.75E-02 | 2.00E-03 |  | 1.35E-02 | 9.90E-03 |
| hip | 1.97E-01 | 1.06E-02 |  | 5.01E-02 | 1.30E-02 |  | 1.28E-02 | 1.70E-03 |  | -6.60E-03 | 9.90E-03 |
| wsthipr | 1.36E-01 | 1.01E-02 |  | 6.08E-02 | 1.55E-02 |  | 9.30E-03 | 1.40E-03 |  | -9.90E-03 | 9.90E-03 |
| diapres | 1.64E-01 | 1.09E-02 |  | 1.66E-02 | 4.60E-03 |  | 1.00E-03 | 9.00E-04 |  | 1.98E-02 | 1.11E-02 |

Note: $\sigma_{g}^{2}$ = phenotypic variance due to genetic effects; $\sigma_{e}^{2}$ = phenotypic variance due to additive effects of exposomic variables; $\sigma_{\mathrm{exe}}^{2}$ = phenotypic variance due to interactions between exposomic variables, and $\sigma_{\mathrm{gxe}}^{2}$ = phenotypic variance due to interactions between genotypes and exposomic variables.

# Supplementary Table 3. P-values for E-C interactions estimated from single-covariate reaction norm models.

| **Trait** | age | sex | pc1 | pc2 | townsend | alc | smk2 | met_all | energy intake |
| --- | --- | --- | --- | --- | --- | --- | --- | --- | --- |
| bmi | 1.31E-02 | 1.34E-02 | 4.00E-01 | 8.91E-02 | 2.43E-05 | 4.33E-22 | 1.65E-32 | 5.59E-21 | 6.80E-08 |
| diapres | 3.37E-12 | 8.31E-02 | 5.76E-01 | 2.64E-01 | 1.60E-01 | 7.25E-01 | 2.56E-01 | 7.96E-02 | 4.91E-01 |
| edu | 2.67E-10 | 2.37E-02 | 3.61E-01 | 5.57E-01 | 1.12E-03 | 2.01E-06 | 1.74E-04 | 9.78E-03 | 3.70E-05 |
| fluidiq | 3.98E-01 | 7.77E-01 | 6.30E-01 | 9.42E-01 | 8.95E-02 | 2.92E-01 | 9.30E-01 | 5.62E-01 | 1.50E-05 |
| heelbmd | 1.04E-03 | 2.55E-02 | 9.53E-02 | 2.46E-01 | 9.36E-01 | 1.14E-02 | 1.78E-01 | 3.70E-03 | 1.10E-01 |
| height | 4.88E-01 | 5.38E-02 | 6.88E-01 | 5.51E-01 | 1.36E-04 | 4.88E-01 | 1.33E-01 | 1.68E-02 | 9.23E-02 |
| hip | 9.92E-03 | 1.88E-02 | 9.44E-01 | 4.34E-01 | 8.89E-04 | 5.80E-13 | 7.00E-17 | 5.74E-24 | 9.92E-06 |
| sitheight | 3.12E-01 | 3.26E-01 | 8.03E-01 | 7.80E-01 | 1.17E-03 | 5.77E-01 | 1 | 2.31E-02 | 1.37E-02 |
| waist | 1.49E-03 | 1.69E-02 | 5.35E-01 | 5.97E-01 | 2.52E-03 | 1.81E-15 | 3.87E-23 | 9.25E-37 | 3.63E-08 |
| weight | 1.24E-03 | 1.73E-02 | 2.08E-01 | 2.68E-02 | 1.56E-04 | 3.31E-16 | 2.44E-26 | 1.26E-23 | 1.89E-07 |
| wsthipr | 5.66E-02 | 1.24E-02 | 5.91E-01 | 4.85E-01 | 1.18E-01 | 1.57E-05 | 7.05E-09 | 1.70E-20 | 2.68E-05 |

Note: 1. univariate reaction norm model: y = g + e_0_ + e_1_*c + ε, where c = covariate (e.g., age). 2. p-values are based on likelihood ratio tests (df=1) that compare the univariate E-C interaction model with a null model (y = g + e_0_ + ε); 3. Highlighted in orange = signals remained after a familywise Bonferroni correction, where a family is defined as 9 univariate analyses for a given trait. Alpha level corrected for multiple testing = 0.05/9 = 5.56E-3.

# Supplementary Table 4. Variance estimates of exc interaction effects from multi-covariates reaction norm models.

| **Trait** | **Covariate** | $\sigma_{\mathrm{exc}}^{2}$ | |
| --- | --- | --- | --- |
|  |  | **estimate** | **s.e.** |
| bmi | townsend | 8.0E-04 | 4.0E-04 |
| bmi | alc | 9.0E-04 | 4.0E-04 |
| bmi | smk2 | 3.0E-03 | 1.0E-03 |
| bmi | met_all | 1.9E-03 | 6.0E-04 |
| bmi | energy | 1.0E-03 | 4.0E-04 |
| diapres | age | 2.0E-03 | 7.0E-04 |
| edu | age | 2.1E-03 | 7.0E-04 |
| edu | townsend | 7.0E-04 | 4.0E-04 |
| edu | alc | 7.0E-04 | 4.0E-04 |
| edu | smk2 | 5.0E-04 | 3.0E-04 |
| edu | energy | 8.0E-04 | 4.0E-04 |
| fluidiq | energy | 1.7E-03 | 8.0E-04 |
| heelbmd | age | 1.4E-03 | 7.0E-04 |
| heelbmd | met_all | 1.4E-03 | 7.0E-04 |
| height | townsend | 9.0E-04 | 4.0E-04 |
| hip | townsend | 6.0E-04 | 3.0E-04 |
| hip | alc | 1.0E-03 | 5.0E-04 |
| hip | smk2 | 1.8E-03 | 7.0E-04 |
| hip | met_all | 2.0E-03 | 7.0E-04 |
| hip | energy | 9.0E-04 | 4.0E-04 |
| sitheight | townsend | 7.0E-04 | 4.0E-04 |
| waist | age | 7.0E-04 | 4.0E-04 |
| waist | townsend | 5.0E-04 | 3.0E-04 |
| waist | alc | 1.0E-03 | 5.0E-04 |
| waist | smk2 | 2.7E-03 | 9.0E-04 |
| waist | met_all | 3.1E-03 | 1.0E-03 |
| waist | energy | 1.2E-03 | 5.0E-04 |
| weight | age | 7.0E-04 | 3.0E-04 |
| weight | townsend | 7.0E-04 | 3.0E-04 |
| weight | alc | 8.0E-04 | 4.0E-04 |
| weight | smk2 | 2.5E-03 | 9.0E-04 |
| weight | met_all | 2.1E-03 | 7.0E-04 |
| weight | energy | 1.0E-03 | 4.0E-04 |
| wsthipr | alc | 5.0E-04 | 3.0E-04 |
| wsthipr | smk2 | 1.2E-03 | 5.0E-04 |
| wsthipr | met_all | 1.9E-03 | 7.0E-04 |
| wsthipr | energy | 1.0E-03 | 5.0E-04 |

# Supplementary Table 5. The correlation structure of exposomic variables affects variance component estimates from the model y = e + exe + ε.

| model parameter |  | true value |  | **uncor. exp.** | |  | **cor.exp.** | |  | **pc** | |
| --- | --- | --- | --- | --- | --- | --- | --- | --- | --- | --- | --- |
|  |  |  |  | ave. | s.e. |  | ave. | s.e. |  | ave. | s.e. |
| $\sigma_{e}^{2}$ |  | 0.4 |  | 0.40 | 0.004 |  | 0.43 | 0.012 |  | 0.40 | 0.003 |
| $\sigma_{\mathrm{exe}}^{2}$ |  | 0.1 |  | 0.10 | 0.002 |  | 0.10 | 0.004 |  | 0.10 | 0.002 |
| $\sigma_{\varepsilon}^{2}$ |  | 0.5 |  | 0.50 | 0.001 |  | 0.50 | 4.5E-04 |  | 0.50 | 0.001 |

Note. 1. uncor. exp.: estimation based on 10 orthogonal exposomic variables simulated from a multivariate normal distribution; cor.exp.: estimation based on 10 correlated exposomic variables simulated from a multivariate normal distribution; pc: estimation based on all principal components of the 10 correlated exposomic variable. 2. The variance estimate of correlated exposomic variables (i.e., $\sigma_{e}^{2}$ under ‘cor.exp.’) can be considered as the estimate from StructLMM^1^. the estimate based on transformed exposomic variables via a principal component analysis (i.e., $\sigma_{e}^{2}$ under ‘pc’) can be considered as the estimate from the proposed integrative analysis of genomic and exposomic data. See supplementary note 1 for the relationship between the two models.

Supplementary Table 6. Exposomic variables used to construct the kernel matrix for estimating exposomic effects on phenotypes.

| **Exposomic Variable** | **Field ID** |
| --- | --- |
| Pack years adult smoking as proportion of life span exposed to smoking | 20162 |
| Alcohol intake (glass & pint/week) | multiple fields |
| MET minutes/week for walking | 22037 |
| MET minutes/week for moderate activity | 22038 |
| MET minutes/week for vigorous activity | 22039 |
| estimated total food weight | 100001 |
| estimated total energy intake | 100002 |
| estimated protein intake | 100003 |
| estimated total fat intake | 100004 |
| estimated carbohydrate intake | 100005 |
| estimated saturated fat intake | 100006 |
| estimated polyunsaturated fat intake | 100007 |
| estimated total sugars intake | 100008 |
| estimated dietary fibre intake | 100009 |
| estimated iron intake | 100011 |
| estimated Vitamin B6 intake | 100012 |
| estimated Vitamin B12 intake | 100013 |
| estimated folate intake | 100014 |
| estimated vitamin C intake | 100015 |
| estimated potassium intake | 100016 |
| estimated magnesium intake | 100017 |
| estimated retinol intake | 100018 |
| estimated carotene intake | 100019 |
| estimated vitamin D intake | 100021 |
| estimated alcohol intake | 100022 |
| estimated starch intake | 100023 |
| estimated calcium intake | 100024 |
| estimated Vitamin E intake | 100025 |

# Supplementary Table 7. Biased estimation of the model y = e + exe + ε can be corrected by removing outliers of exposomic variables and performing a principal component analysis on exposomic variables.

| model parameter |  | true value |  | **before** | |  | **pc** | |  | **qc** | |  | **pc + qc** | |
| --- | --- | --- | --- | --- | --- | --- | --- | --- | --- | --- | --- | --- | --- | --- |
|  |  |  |  | ave. | s.d. |  | ave. | s.d. |  | ave. | s.d. |  | ave. | s.d. |
| $\sigma_{e}^{2}$ |  | 0.4 |  | 0.49 | 0.22 |  | 0.42 | 0.06 |  | 0.48 | 0.23 |  | 0.40 | 0.04 |
| $\sigma_{\mathrm{exe}}^{2}$ |  | 0.1 |  | 0.08 | 0.04 |  | 0.09 | 0.02 |  | 0.10 | 0.04 |  | 0.10 | 0.01 |
| $\sigma_{\varepsilon}^{2}$ |  | 0.5 |  | 0.50 | 4.9E-03 |  | 0.50 | 0.01 |  | 0.50 | 0.01 |  | 0.50 | 0.01 |

Note. before: estimation based on ten exposomic variables from the UK biobank, which are correlated and have skewed distributions; pc: estimation based on all principal components of the exposomic variables; qc: estimation based on quality-controlled exposomic variables, of which values outside +/-3 sd from the mean are removed; pc + qc: estimation based on all principal components of the quality controlled exposomic variables.

# Supplementary Table 8. Simulation results based on 10 real correlated exposomic variables under the model y = g + e + gxe + ε when $\sigma_{\mathrm{gxe}}^{2}$ is relatively large.

| model parameter |  | true value |  | **before** | |  | **pc1** | |  | **pc2** | |
| --- | --- | --- | --- | --- | --- | --- | --- | --- | --- | --- | --- |
|  |  |  |  | ave | sd |  | ave | sd |  | ave | sd |
| $\sigma_{g}^{2}$ |  | 0.3 |  | 0.28 | 0.08 |  | 0.27 | 0.09 |  | 0.28 | 0.08 |
| $\sigma_{e}^{2}$ |  | 0.3 |  | 0.35 | 0.16 |  | 0.30 | 0.02 |  | 0.30 | 0.02 |
| $\sigma_{\mathrm{gxe}}^{2}$ |  | 0.3 |  | 0.27 | 0.11 |  | 0.30 | 0.17 |  | 0.27 | 0.11 |
| $\sigma_{\varepsilon}^{2}$ |  | 0.1 |  | 0.13 | 0.11 |  | 0.12 | 0.15 |  | 0.13 | 0.11 |

Note: before: estimation based on the original exposomic variables; pc1: the erm is constructed using the principal components (PCs) of the exposomic variables, and the kernel matrix for gxe is based on the hadamard product of the grm and the erm constructed using PCs of the exposomic variables; pc2: the erm is constructed using the principal components of the exposomic variables, and the kernel matrix for gxe is based on the hadamard product of the grm and the erm constructed using original exposomic variables.

# Supplementary Table 9. Simulation results based on 10 real correlated exposomic variables under the model y = g + e + gxe + ε when $\sigma_{\mathrm{gxe}}^{2}$ is relatively small.

| model parameter |  | true value |  | **before** | |  | **pc1** | |  | **pc2** | |
| --- | --- | --- | --- | --- | --- | --- | --- | --- | --- | --- | --- |
|  |  |  |  | ave | sd |  | ave | sd |  | ave | sd |
| $\sigma_{g}^{2}$ |  | 0.3 |  | 0.29 | 0.06 |  | 0.28 | 0.06 |  | 0.29 | 0.06 |
| $\sigma_{e}^{2}$ |  | 0.3 |  | 0.35 | 0.15 |  | 0.30 | 0.01 |  | 0.30 | 0.01 |
| $\sigma_{\mathrm{gxe}}^{2}$ |  | 0.1 |  | 0.09 | 0.07 |  | 0.11 | 0.11 |  | 0.09 | 0.07 |
| $\sigma_{\varepsilon}^{2}$ |  | 0.3 |  | 0.31 | 0.07 |  | 0.29 | 0.11 |  | 0.31 | 0.07 |

Note: before: estimation based on the original exposomic variables; pc1: the erm is constructed using the principal components (PCs) of the exposomic variables, and the kernel matrix for gxe is based on the hadamard product of the grm and the erm constructed using PCs of the exposomic variables; pc2: the erm is constructed using the principal components of the exposomic variables, and the kernel matrix for gxe is based on the hadamard product of the grm and the erm constructed using original exposomic variables.

Supplementary Table 10. Simulation results based on 10 correlated exposomic variables.

| model parameter |  | true value |  | **before** | |  | **pc1** | |  | **pc2** | |
| --- | --- | --- | --- | --- | --- | --- | --- | --- | --- | --- | --- |
|  |  |  |  | ave | sd |  | ave | sd |  | ave | sd |
| $\sigma_{g}^{2}$ |  | 0.3 |  | 0.30 | 0.05 |  | 0.30 | 0.05 |  | 0.30 | 0.05 |
| $\sigma_{e}^{2}$ |  | 0.3 |  | 0.32 | 0.09 |  | 0.30 | 0.01 |  | 0.30 | 0.01 |
| $\sigma_{\mathrm{gxe}}^{2}$ |  | 0.3 |  | 0.28 | 0.06 |  | 0.30 | 0.05 |  | 0.28 | 0.06 |
| $\sigma_{\varepsilon}^{2}$ |  | 0.1 |  | 0.12 | 0.07 |  | 0.10 | 0.06 |  | 0.12 | 0.07 |

Note: Exposomic variables were simulated using a multivariate normal distribution with a variance and covariance matrix that contains non-zero off-diagonal entries. before: estimation based on the simulated exposomic variables without any transformation; pc1: the erm is constructed using the principal components (PCs) of the exposomic variables, and the kernel matrix for gxe is based on the hadamard product of the grm and the erm constructed using PCs of the exposomic variables; pc2: the erm is constructed using the principal components of the exposomic variables, and the kernel matrix for gxe is based on the hadamard product of the grm and the erm constructed using original exposomic variables.

# **Supplementary Note 1**

Here we elaborate on the equivalence of the proposed integrative analysis of genomic and exposomic data (IGE) with StructLMM.

Quoted from Moore et al.^1^, StructLMM expresses the phenotypes of a complex trait, **y**, as

$\mathbf{y}= \mathbf{Xb} + \mathbf{x}\beta_{G} +\sum_{l=1}^{L} (\mathbf{x} \otimes\mathbf{e}_{\mathbf{l}})\beta_{l} + \sum_{l=1}^{L} \mathbf{e}_{\mathbf{l}}\alpha_{l} + \boldsymbol{\Psi}$ (1)

$$\mathbf{e}$$

$$\boldsymbol{\varepsilon}$$

$$\boldsymbol{g\times e}$$

$$\mu\mathbf{I}$$

where $\beta_{l} \sim N (\mathbf{0}, {\sigma_{\mathrm{gxe}}^{2}}/L)$, $\alpha_{l} \sim N (\mathbf{0}, {\sigma_{e}^{2}}/L)$, and $\Psi\sim N (\mathbf{0}, \sigma_{\varepsilon}^{2}\mathbf{I})$.

Here $\mathbf{Xb}$ is the fixed effects of covariates on phenotypes, **x** stores genotypes of a given SNP, $\beta_{G}$ is the fixed effect or persistent effect of the SNP on phenotypes, and $\mathbf{e}_{\mathbf{1}}$, $\mathbf{e}_{\mathbf{2}}$, … $\mathbf{e}_{\mathbf{L}}$ are the set of standardized values of environments 1, 2 to L. $\beta_{l}$ is the random effect of SNP x environment l interaction with a variance ${\sigma_{\mathrm{gxe}}^{2}}/L$, $\alpha_{l}$ is the random effect of environment l with a variance ${\sigma_{e}^{2}}/L$, and $\Psi$ contains residuals with a variance $\sigma_{\varepsilon}^{2}$.

The IGE equivalent terms in Equation (1) are highlighted in the annotations. Importantly, the g x e term in StructLMM is for a single SNP, but in IGE it is aggregated across all common SNPs of the genome. Under the null hypothesis, i.e., $\sigma_{\mathrm{gxe}}^{2}$=0, StructLMM reduces to

$\mathbf{y}= \mathbf{Xb} + \mathbf{x}\beta_{G} + \sum_{l=1}^{L} \mathbf{e}_{\mathbf{l}}\alpha_{l} + \boldsymbol{\Psi}$ (2)

$$\boldsymbol{\varepsilon}$$

$$\mathbf{e}$$

$$\mu\mathbf{I}$$

Equation (2) becomes equivalent to a simplified IGE that assumes $\sigma_{g}^{2}=0$ (see Equation 2a in Table 2).

**References**

1. Moore, R. *et al.* A linear mixed-model approach to study multivariate gene–environment interactions. *Nature Genetics* **51**, 180-186 (2019).
